# Decline in gut motility of cerebral palsy patients after a triggering event: A discussion on invasive versus conservative management

**DOI:** 10.1002/jpr3.70076

**Published:** 2025-08-17

**Authors:** Zoe Saenz, Elizabeth Reynolds, Jamie E. Anderson, Payam Saadai, Maheen Hassan

**Affiliations:** ^1^ Department of Surgery University of California, Davis Children's Hospital Sacramento California USA; ^2^ Department of Pediatrics University of California, Davis Children's Hospital Sacramento California USA

**Keywords:** case series, dysmotility, pediatrics, Pediatric Intestinal Pseudo‐Obstruction (PIPO), quality of life

## Abstract

**Objectives:**

Patients with cerebral palsy (CP) often have gastrointestinal dysmotility. An inciting event, such as infection, may lead to progressive decline in bowel motility and episodes of acute pediatric intestinal pseudo‐obstruction (PIPO). Surgery can be implemented when medical therapy fails, but it is unclear if it can improve or lengthen the quality of life. Here, we explore this question with a case series.

**Methods:**

We performed a retrospective chart review and identified five patients with CP who were hospitalized between January 2017 to January 2024, secondary to a triggering event. They all developed subsequent decline in bowel function.

**Results:**

We present five gastrostomy tube‐dependent patients with CP who had prolonged hospitalizations after a triggering event and an associated decline in intestinal motility. Case 1 is a 7‐year‐old female with feeding intolerance after a viral infection and a hospital stay of 30 days. She received anal sphincter botulinum toxin injection and returned to baseline. Case 2 is 21‐year‐old male with aspiration pneumonia who became dependent on total parenteral nutrition (TPN). He died after complications associated with midgut volvulus. Case 3 is an 18‐year‐old male with feeding intolerance following COVID and required several procedures, a hospital stay of 205 days, and gradual return to baseline. Case 4 is a 15‐year‐old male with a small bowel obstruction and recurrent volvulus, prolonged hospitalization, and death. Case 5 is a 4‐year‐old female with frequent PIPO triggered by urinary tract infections.

**Conclusion:**

Patients with CP are susceptible to a decline in bowel function. A balance between prolonging life and improving quality of life should always be considered. TPN in place of surgical interventions might help decrease hospitalizations and surgical morbidity. Surgery is reasonable for mechanical obstruction, but invasive procedures should not be assumed to improve quality of life.

## Abstract (highlights)

**What is Known:**

- Patients with cerebral palsy (CP) have intestinal dysmotility.
- Management of pediatric intestinal pseudo‐obstruction includes bowel rest and nutritional support.

**What is New:**

- A triggering event may lead to decline in intestinal motility in patients with CP.
- Surgical decompression should not be assumed to increase quality of life or prolong life in this population.

## 1 INTRODUCTION

Cerebral palsy (CP) is a spectrum of neurological disorders characterized by impaired movement, posture, and potential developmental delay.^1^ CP occurs after an injury to the developing brain in the prenatal or neonatal period with an overall prevalence of 1.5–3 per 1000 live births in the United States.^2^ This disease process has heterogenous neuromotor manifestations and requires lifelong multidisciplinary care. Among these neuromotor manifestations are challenges with bowel motility.

Children with CP have compromised neuronal, hormonal, and biohumoral interactions between the gut and brain. For example, the rate of chronic constipation in this patient population is estimated to be as high as 74%.^3^ Furthermore, this population achieves bladder and bowel continence at an older age, if at all, when compared to peers without CP. In fact, only 50% of CP patients were continent at age 11 in a population‐based study.^4^ The reasons for this are multifactorial, owing to cognitive and motor disabilities, as well as a spectrum of complexities including neurogenic bowel and enteric neuropathy. Studies suggest the prevalence of digestive comorbidities such as gastroesophageal reflux disease (GERD) and delayed gastric emptying are associated with the severity of CP.^5^ Consequently, patients with severe, spastic CP may be uniquely susceptible to pediatric intestinal pseudo‐obstruction (PIPO).^3^, ^5^

In PIPO, patients present with clinical features of bowel obstruction without a mechanical etiology. Autoimmune, infectious, endocrine, and developmental conditions have all been associated with PIPO, amongst an extensive list of secondary causes.^5^ The diagnosis of PIPO is based on meeting at least two of the four following criteria: (1) Objective measurement of small intestinal neuromuscular involvement based on manometry, histopathology, or transit. (2) Recurrent and/or persistently dilated loops of small intestine on imaging. (3) Genetic and/or metabolic abnormalities definitively associated with PIPO. (4) Inability to maintain adequate nutrition and/or growth on oral feeds, necessitating enteral or parental nutrition support.^6^ Treatment focuses on optimizing nutrition and preserving bowel function. When conservative therapy fails, surgical intervention may be a reasonable step in management yet remains controversial because its benefits in patients with PIPO are unclear.^7^

Here, we present a case series of five patients with CP who had a decline in bowel function after a triggering event. We explore the role of decompressive therapy when medical management has failed and its impact on future bowel function and quality of life. The aim of this case series is to provoke discussion regarding conservative versus invasive interventions for PIPO in CP patients. To our knowledge, this is the first case series considering this important question. This manuscript was prepared using CARE guidelines.

## 2 METHODS

We performed a retrospective chart review and identified five patients with CP who were hospitalized between January 2017 to January 2024, secondary to a triggering event. They all developed subsequent decline in bowel function, defined as intolerance to enteral nutrition without the ability to return to baseline feeds. Prolonged hospitalization was defined as more than 30 days. All five patients were gastrostomy tube dependent and tolerating feeds before their sentinel event and met criteria for PIPO during a subsequent admission. We reviewed their relevant history, presenting symptoms, laboratory and radiographic work‐up, hospital course, procedures, re‐admissions, and follow‐up information.

### 2.1 Ethics statement

This study was conducted in compliance with the University of California, Davis Institutional Review Board.

## 3 RESULTS

### 3.1 Case 1

**Figure 1:**
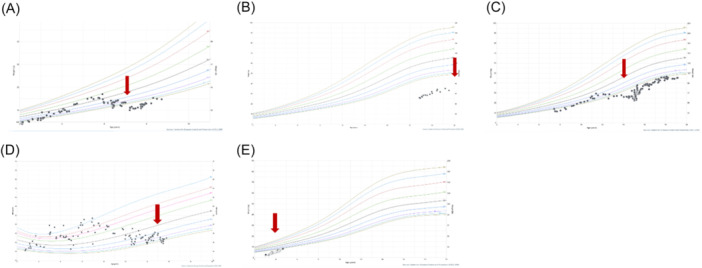
Growth curves for case 1 (A), case 2 (B), case 3 (C), case 4 (D), case 5 (E), with red arrows indicating each patient's triggering event.

Case 1 is a 7‐year‐old girl with spastic quadriplegia, developmental delay, and hydrocephalus with a ventriculoperitoneal shunt. Her baseline out‐patient bowel regimen consisted of small volume enemas, polyethylene glycol 3350, and senna. At that time, she was on a regimen of continuous enteral feeds with some per oral intake. Additional co‐morbidities are outlined in Table 1. One month following a viral illness, she was admitted with feeding intolerance, abdominal distension, and a computed tomography (CT) abdomen pelvis consistent with ileus. Her home enteral feeds were held, and she was trialed on bowel rest with both gastrostomy tube to gravity drainage and rectal tube decompression. During the same hospital admission, she underwent anal sphincter botulinum toxin injection with initiation of erythromycin and metoclopramide. She was diagnosed with PIPO given that she had a 17% weight loss relative to her admission weight and persistently dilated bowel on imaging. Furthermore, on admission, she was tracking along the fifth percentile and was subsequently off the curve for more than 1 year (Figure 1A). She was unable to return to gastric feeds and required transition to jejunal feeding. She no longer tolerated any oral intake as she had before this admission (Table 2). Total hospital length of stay of 30 days.

**Table 1:** Patient characteristics and baseline medical management.

Patient	Comorbidities	Baseline medical management	Baseline nutrition
1 (7 yo, F) spastic quadriplegia	Developmental delay, hypoxic‐ischemic encephalopathy s/p ventriculoperitoneal shunt, seizures, chronic lung disease, asthma, obstructive sleep apnea, hypertrophic cardiomyopathy, pulmonary hypertension	Enemas, polyethylene glycol, senna	Continuous gastric feeds with 2 h. breaks every 4 h. Occasional small volume PO.
2 (21 yo, M, deceased) spastic quadriplegia	Developmental delay, seizures, gastroesophageal reflux disease	Erythromycin, metoclopramide, prucalopride	Bolus gastric feeds. No PO.
3 (19 yo, M) spastic quadriplegia	Developmental delay, epilepsy, tetralogy of Fallot s/p repair, scoliosis	Amoxicillin/clavulanate	Bolus gastric feeds. Occasional small volume PO.
4 (15 yo, M, deceased) spastic quadriplegia	Developmental delay, Pierre‐Robinson syndrome, atrial septal defect s/p repair, metotropic craniosynostosis s/p cranioplasty, laryngotracheomalacia, tethered cord	Metoclopramide	Longer jejunal feeding intervals instead of bolus feeds. No PO.
5 (4 yo F) spastic dystonic	Seizure disorder, leukodystrophy, recurrent urinary tract infections	Senna, simethicone, miralax, prucalopride	Bolus gastric feeds. Occasional PO.

Abbreviations: F, female; M, male; PO, per ora; S/p, status post.

**Table 2:** Number of procedures, procedures performed, length of stay and nutrition plan after triggering event.

Patient	No. procedures	Triggering event	Procedures	Admissions	LOS (days) after triggering event	Nutrition after triggering event
1	2	Viral illness	1. Anal sphincter botulinum toxin injection. 2. Gastrostomy tube to gastro‐jejunal tube conversion.	4	30	Gastro‐jejunal feeds. No PO.
2	3	Aspiration pneumonia	1. Anal sphincter botulinum toxin injection, rectal biopsy. 2. Laparoscopic loop ileostomy. 3. Exploratory laparotomy, small bowel resection and end ileostomy.	9	167	TPN, no tolerance of even small volume enteral feeds. No PO.
3	3	COVID	1. Gastrostomy tube to gastrojejunostomy conversion. 2. Nissen fundoplication, Roux‐en‐Y gastrojejunostomy, revision of gastrostomy. 3. Closure of gastrocutaneous fistula, replacement of gastrostomy tube.	8	205	Jejunal feeds, supplemental TPN. No PO.
4	3	Urinary tract infection	1. Exploratory laparotomy and lysis of adhesions. 2. Exploratory laparotomy and lysis of adhesions for recurrent volvulus. 3. Exploratory laparotomy Roux‐en‐Y. gastrojejunostomy, lysis of adhesions.	21	138	TPN only.
5	2	Recurrent urinary tract infections	1. Gastrostomy tube placement. 2. Anal sphincter botulinum toxin.	5	109	Gastric feeds Supplemental TPN. No PO.

Abbreviations: LOS, length of stay; No., number; PO, per oral; TPN, total parenteral nutrition.

### 3.2 Case 2

Case 2 is a 21‐year‐old, now deceased male with spastic quadriplegia, developmental delay, seizures, and GERD with a surgical history of a Nissen fundoplication. His outpatient medical management consisted of erythromycin and metoclopramide to promote motility and received bolus feeds via gastrostomy tube with no oral intake. After a bout of aspiration pneumonia, he presented with abdominal distension and feeding intolerance. CT abdomen pelvis with intravenous contrast was notable for overall dilated colon and areas of focal narrowing in the rectosigmoid colon. He thus underwent a contrast enema and flexible sigmoidoscopy, both of which ruled out the presence of stricture. Rectal biopsies were also obtained and ruled out Hirschsprung disease. His presentation was overall consistent with chronic colonic dysmotility. Similarly, 3 years prior, he had been admitted with PIPO and had improved with anal sphincter botulinum toxin injection, but at this presentation he failed a trial of intravenous neostigmine and senna, and did not improve with anal sphincter botulinum toxin injection. He was ultimately discharged with a plan for prolonged bowel rest on TPN. One month later, he presented with worsening abdominal distension. Admission CT abdomen pelvis was notable for dilated small bowel and colon without evidence of obstruction. After a multidisciplinary discussion, he underwent a laparoscopic loop ileostomy for diversion in the setting of continued chronic colonic dilation and dysmotility. Postoperatively, he had some improvement in symptoms and began tolerating small enteral feeds with supplemental TPN. He was discharged home and readmitted 3 months later with aspiration pneumonia in the setting of a seizure event, requiring intubation and vasopressor support. He recovered from his respiratory event and was maintained on minimal tube feeds with supplemental TPN. However, this inciting event was soon followed by progressive agitation, feeding intolerance, and a precipitous decline in bowel function within 1 month. Admission CT abdomen/pelvis was suspicious for midgut volvulus for which he underwent an emergent laparotomy with small bowel resection and end ileostomy. The total hospital length of stay was 167 days after his inciting event, which we determined to be aspiration pneumonia. After this final procedure, the patient remained intubated and was ultimately transitioned to comfort care by his caretakers.

### 3.3 Case 3

Case 3 is a now 19‐year‐old male with spastic quadriplegia, developmental delay, and epilepsy. Outpatient bowel management consisted of only amoxicillin/clavulanic acid for small bowel dysmotility. Following a COVID infection (age 14), he had a gradual decline in tolerance of gastric feeds and weight loss and was admitted three months later with aspiration pneumonia. He had previously been tracking along the tenth percentile for weight and had fallen to the first percentile (Figure 1C). His symptoms were attributed to PIPO given persistently dilated bowel on imaging and inability to maintain nutrition on enteral feeds. To aid in feeding tolerance, his gastrostomy tube was transitioned to a gastro‐jejunal (GJ) tube during this admission. He had recurrent episodes of the GJ tube coiling in the stomach, thought to be due to an abnormally narrowed distal duodenum. A multidisciplinary meeting with the family discussed the need for either long‐term TPN versus surgical management, given that he had not had substantial improvement in small gastrointestinal dysmotility with amoxicillin/clavulanic acid and the challenges with the GJ tube. His family opted to proceed with surgical management, and he underwent a Nissen fundoplication for recurrent aspiration, Roux‐en‐Y gastrojejunostomy with jejunostomy tube for permanent feeding access, and revision of gastrostomy tube for gastric decompression. Despite this, he continued to have feeding intolerance and gastrostomy tube leakage. He was ultimately discharged on trophic feeds with supplemental TPN. His total length‐of‐stay (LOS) after the sentinel event, COVID infection, was 205 days. He has very slowly regained weight and finally was back on the fifth percentile of growth curve nearly 3 years later.

### 3.4 Case 4

Case 4 is a now deceased 15‐year‐old male with spastic quadriplegia and a history of chromosome 9p deletion, Pierre‐Robinson syndrome, atrial septal defect with prior repair, metotropic craniosynostosis and history of cranioplasty, laryngotracheomalacia, and tethered cord. He therefore had multiple surgical and anesthetic events starting at an early age, including a Ladds procedure as an infant and exploratory laparotomy for colonic volvulus with lysis of adhesions only (at age 11), and planned exploratory laparotomy with subtotal colectomy at age 12 to prevent recurrence of future episodes of volvulus. At baseline, he was managed with jejunal feeds and metoclopramide, and was tracking between the 10th and 25th percentile growth curve. At age 15, he presented with constipation and emesis, overall concerning for partial small bowel obstruction versus ileus. He was also noted to have a urinary tract infection and admission abdominal X‐ray notable for gastro‐jejunostomy (GJ) tube coiled in the stomach. Of note, his GJ tube had required repositioning by interventional radiology on several occasions. During this admission he had continued weight loss, feeding intolerance, high gastrostomy tube output, ileus, and need for TPN. By this time, he had fallen below the 3rd percentile on the growth curve (Figure 1D). He underwent an elective exploratory laparotomy with lysis of adhesions without evidence of mechanical obstruction and Roux‐en‐Y gastro‐jejunostomy for permanent jejunal feeding access. He was discharged tolerating jejunal feeds. Unfortunately, he developed a high‐grade bowel obstruction 1 month later. He was transitioned to comfort care after discussing with his family that further invasive operations would be unlikely to improve his quality of life.

### 3.5 Case 5

**Figure 2:**
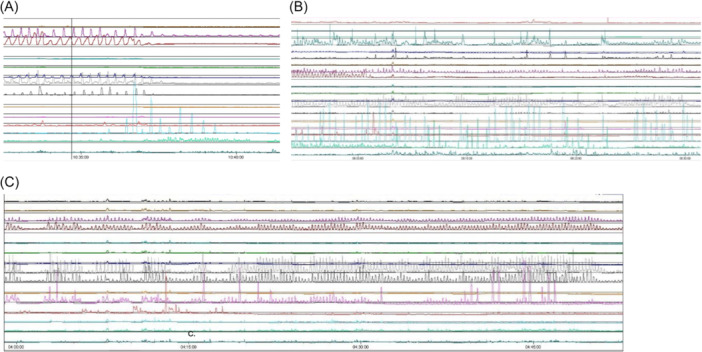
Case 5 antroduodenal manometry. (A) Fasting phase. Phase III MMC is shown with antrum contracting at a normal frequency and robust amplitude, small bowel contracting at a normal frequency but low amplitude. (B) Posterythromycin. There is an increase primarily in pyloric contractions and amplitude. (C) Postprandial. Similar to the fasting state, there is a very active antrum but the small bowel does not increase in activity. Not pictured here: return of phase III MMC within 60 min of meal. MMC, migrating motor complex.

Case 5 is a 4‐year‐old female with spastic dystonic CP, leukodystrophy, seizure disorder, and neurogenic bladder with recurrent urinary tract infections. At baseline, she was receiving enteral feeds through a nasogastric tube for nutrition and had been persistently below the growth curve for her age (Figure 1E). She was admitted with fever and abdominal distension in the setting of a recently treated urinary tract infection. CT abdomen pelvis was obtained demonstrating acute pyelonephritis. Her imaging was also notable for small bowel dilation concerning for post‐infectious ileus. In the setting of her failure to thrive, she additionally underwent a gastrostomy tube placement for long‐term nutrition and anal sphincter botulinum toxin injection for chronic constipation. She was discharged tolerating gastrostomy tube feeds after recovering from her acute illness. Unfortunately, she was readmitted 6 weeks later with vomiting and constipation. At that time, her mother reported that the botulinum toxin injection from her prior admission had not noticeably helped her constipation. An abdominal X‐ray demonstrated rectal stool but otherwise indicated a nonobstructive pattern suspicious for recurrent PIPO. She improved with a low‐volume saline enema and increased regimen of polyethylene glycol and senna. She was discharged tolerating gastrostomy tube feeds. She was once again admitted with a similar presentation 2 months later. With persistent feeding intolerance now over the course of several months, she was started on TPN and her gastrostomy tube was converted to a gastro‐jejunostomy tube. She underwent antroduodenal and colonic manometry testing as well as repeat anal sphincter botulinum toxin injection. Antroduodenal manometry showed normal migrating motor complex (MMC) architecture during fasting phase and appropriate response to erythromycin stimulation with increased antral contractions (Figure 2). However, the small bowel contracted at a low amplitude throughout the study and there was impaired response to a meal, cycling through an MMC during the 1 h postprandial window. Colonic manometry was normal. Given these findings, she was started on prucalopride and amoxicillin/clavulanic acid with significant improvement in her feeding tolerance and was discharged home on continuous gastric feeds. Unfortunately, since this time, she has struggled to stay out of the hospital for more than 2 weeks. She was readmitted four more times, three of which were due to a UTI with resulting post‐infectious dysmotility. Her most recent admission was for status epilepticus and autonomic storm, and her hospitalization has been prolonged due to dysmotility requiring TPN.

## 4 DISCUSSION

CP is a complex multisystem disorder. A seemingly low‐risk event, such as a viral or bacterial illness, may have serious long‐term ramifications with respect to gut motility in this highly susceptible population. In our case series, these sentinel events included triggers such as viral illnesses, urinary tract infections, and aspiration pneumonia. In each case, even if this sentinel event was not primarily an abdominal disease process, the patients developed feeding intolerance with acute or acute‐on‐chronic PIPO as part of the differential diagnosis.

While we assume that the underlying gastrointestinal dysmotility in these patients is secondary to their CP diagnosis, this association has yet to be elucidated or fully understood. Our case series is consistent with existing literature, which primarily consists of only a few case reports and observational studies.^7^, ^8^, ^9^, ^10^ For example, Dewey et al presented the case of a 19‐year‐old patient with CP who developed feeding intolerance and sudden cardiac arrest shortly thereafter.^7^, ^11^ The autopsy findings were concerning for acute colonic pseudo‐obstruction so severe that it led to lung compression and subsequent cardiovascular collapse. In patients with developmental delay, characterizing abdominal pain might be challenging given that patients may not articulate their pain, leading to a delay in diagnosis. This case emphasizes the indolent presentation yet severe ramifications of PIPO in an already fragile and susceptible population.

Consensus guidelines from the European Society for Pediatric Gastroenterology Hepatology and Nutrition (ESPGHAN) outline recommendations for management of suspected PIPO.^12^ Management begins with optimizing nutrition, with parenteral nutrition if necessary, and utilizing promotility agents. If medical management fails, it is suggested to proceed with surgical interventions such as gastrostomy for feeding and venting and ileostomy versus colostomy for decompression, as these interventions have largely shown to benefit feeding tolerance. Similar practice guidelines exist for pseudo‐obstruction in for adults, but there is little evidence if these practice guidelines equally apply to those with underlying neurological disorders like CP.

In the setting of suspected PIPO, there may be a discussion of conservative versus invasive management, depending on the time course and severity of presentation. TPN may be used as a bridge to enteral feeding in patients with acute pseudo‐obstruction. In some cases, however, it is unclear if TPN will be temporary or if a patient's potentially progressive gut failure will lead to lifelong dependence on parenteral nutrition. Manometry can be used to help delineate the location as well as severity of dysmotility. Information gained from this testing can help guide whether TPN should be initiated. Currently, both clinical and manometry findings are used to determine the need for TPN. While there is no consensus or data on what manometry findings predict TPN need, there is a single paper that found that patients with no phase III MMC within 4 h were more likely to require TPN. Great Ormond Street Hospital London ADM Scoring System (GLASS), was published recently and gives a score based on characteristics of each phase in the MMC cycle as well as postprandial state.^13^ The authors of the GLASS score demonstrated a better correlation between abnormal histopathology and the GLASS score versus that of conventional analysis. They also found that a score >13 was predictive of TPN need. Larger studies are needed using this scoring system to better assess clinical application.

Before our patient in case 5 underwent antroduodenal and colonic manometry, there was excessive attention directed at promoting colonic motility due to the mother's perception that the patient's distention and discomfort was due to the inability to stool. Determining that her colonic motility was normal and her small bowel was the underlying issue helped focus our medical interventions at optimizing her small bowel motility, treating small intestine bacterial overgrowth, and managing the visceral hyperalgesia from her small bowel distention. It also helped with the decision to provide long‐term TPN when all medical interventions failed.

Abdominal surgery may be performed in cases when mechanical obstruction must be treated or excluded. PIPO may have mechanical sequelae such as volvulus from severe bowel dilation, as observed in our series. However, chronic dysmotility in the setting of an extensive surgical history may prove to be a diagnostic challenge. Additionally, patients may benefit from a proximal diversion as it aids in decompression and can allow patients to advance enteral feeds. Patient 2 benefited from an ileostomy, as it relieved him from his severe abdominal distention and allowed him to tolerate medications and trophic feeds enterally after proximal diversion. It is difficult to determine if this intervention extended his life expectancy, as he had complications that led to his death shortly after. In our case series, surgical intervention that did not serve as a mode of decompression did not necessarily improve outcomes. Patients 3 and 4 both underwent Roux‐en‐Y gastrojejunostomies and had prolonged hospitalizations after surgery. It is difficult to know if the one patient who returned to enteral feeds could have had the same outcome with bowel rest and TPN alone.

Quality of life outcomes should be carefully considered before operative intervention. In this case series, invasive interventions such as exploratory laparotomy contributed to progression to comfort care in two patients. However, in a third case, the patient gradually returned to baseline, albeit after more than 200 days in the hospital and many invasive procedures. In the end, he was discharged on TPN despite these operations which may have prolonged his length of stay by several months. Importantly, we must consider risks associated with prolonged TPN use such as central line infection and hepatic dysfunction.^14^ In each case, we recommend an individualized approach, thoughtful family counseling, and a multidisciplinary discussion with gastroenterology, surgery, social work, and palliative care if available.

Finally, it should be noted that while we emphasize the association of a tenuous nature of a CP individual's motility, the gut‐brain interaction is bidirectional. Therefore, a decline in intestinal motility may be associated with a decline in neurological function.^15^ We have observed this finding in case 5, who has since had worsening of her seizure disorder with subsequent admissions for status epilepticus.

Our study is most limited by its small sample size and retrospective study design. A larger randomized or prospective study would be ethically challenging in an already tenuous management situation and fragile population. A long‐term follow‐up or larger cohort study may help to better characterize the impact of PIPO in CP patients, particularly in those without an abdominal surgical history that might confound the picture. For example, patient 4 had an extensive surgical history, and it is challenging to definitively rule out any component of surgical adhesive disease playing a role in his presentation. Furthermore, his final operation was done at an outside hospital for which we do not have records for intra‐operative findings. To illustrate a case without a complex prior surgical history, we included patient 5 who presented with recurrent pseudo‐obstructions even before the placement of her gastrostomy tube, which was her only abdominal surgery. Together, these cases illustrate the diversity of this complicated disease process and the need for thoughtful consideration about long‐term management in CP patients with chronic dysmotility.

## 5 CONCLUSION

CP patients are uniquely susceptible to a decline in bowel function. This decline might be associated with an identifiable triggering event. In a neurodiverse individual, clinicians should maintain a high suspicion for PIPO and consider the possible associated ethical dilemmas with respect to intervention. Long‐term quality of life outcomes are difficult to predict but should be openly discussed when counseling these families in a multidisciplinary setting. Careful consideration as to whether an invasive intervention is beneficial when considering quality of life outcomes for the patient is paramount.

## CONFLICT OF INTEREST STATEMENT

The authors declare no conflicts of interest.
